# A large and diverse collection of bovine genome sequences from the Canadian Cattle Genome Project

**DOI:** 10.1186/s13742-015-0090-5

**Published:** 2015-10-26

**Authors:** Paul Stothard, Xiaoping Liao, Adriano S. Arantes, Mary De Pauw, Colin Coros, Graham S. Plastow, Mehdi Sargolzaei, John J. Crowley, John A. Basarab, Flavio Schenkel, Stephen Moore, Stephen P. Miller

**Affiliations:** 1 Department of Agricultural, Food and Nutritional Science / Livestock Gentec, University of Alberta, Edmonton, AB Canada; 2 Tianjin Institute of Industrial Biotechnology, Chinese Academy of Sciences, Tianjin, China; 3 Delta Genomics, Edmonton, AB Canada; 4 Queensland Alliance for Agriculture & Food Innovation, University of Queensland, St Lucia, Australia; 5 Centre for Genetic Improvement of Livestock, University of Guelph, Guelph, ON Canada; 6 Alberta Agriculture, Food and Rural Development, Lacombe Research Centre, Lacombe, AB Canada; 7 AgResearch Limited, Invermay Agricultural Centre, Mosgiel, New Zealand

**Keywords:** Whole-genome sequencing, *Bos taurus*, Beef, Dairy

## Abstract

**Background:**

The Canadian Cattle Genome Project is a large-scale international project that aims to develop genomics-based tools to enhance the efficiency and sustainability of beef and dairy production. Obtaining DNA sequence information is an important part of achieving this goal as it facilitates efforts to associate specific DNA differences with phenotypic variation. These associations can be used to guide breeding decisions and provide valuable insight into the molecular basis of traits.

**Findings:**

We describe a dataset of 379 whole-genome sequences, taken primarily from key historic *Bos taurus* animals, along with the analyses that were performed to assess data quality. The sequenced animals represent ten populations relevant to beef or dairy production. Animal information (name, breed, population), sequence data metrics (mapping rate, depth, concordance), and sequence repository identifiers (NCBI BioProject and BioSample IDs) are provided to enable others to access and exploit this sequence information.

**Conclusions:**

The large number of whole-genome sequences generated as a result of this project will contribute to ongoing work aiming to catalogue the variation that exists in cattle as well as efforts to improve traits through genotype-guided selection. Studies of gene function, population structure, and sequence evolution are also likely to benefit from the availability of this resource.

**Electronic supplementary material:**

The online version of this article (doi:10.1186/s13742-015-0090-5) contains supplementary material, which is available to authorized users.

## Data description

### Animal selection

The sequencing dataset presented here primarily consists of key influential sires of ten cattle populations. Seven of these are the Simmental, Limousin, Angus, Charolais, Hereford, Gelbvieh, and Holstein purebreds. Holstein is an important dairy breed, whereas the others are used in beef production. The remaining three populations, referred to as Alberta, Guelph, and Beefbooster, are crossbred and composite animals with purebred, crossbred or composite sires. The Alberta population consists of research animals located at the Agriculture and Agri-Food Canada research station at Lacombe, and the University of Alberta research station at Kinsella, Alberta, Canada. Sires of these animals are mostly purebred (all breeds mentioned previously plus Red Angus) and Beefbooster animals (see below). Three of the sequenced sires are referred to as Kinsella composites (KC) of many different breeds, created at the station in the 1960s. The Guelph population consists of animals in the University of Guelph beef research herd, located at New Liskeard and Elora, Ontario, Canada. Although the herds have origins going back to the 1970s, since 1995 Angus (black and red) and Simmental sires, or sires that are a combination of these breeds (composite or hybrid sires) have predominantly been used. These research populations usually have recorded phenotypes such as feed efficiency, and have been largely selected in parallel with commercial selection trends. Beefbooster is a population (including the sequenced sires) of hybrid animals created in the 1970s. They are a composite of many other breeds including Angus, Simmental, Gelbvieh, Limousin, and Shorthorn. The purpose of creating this population was to capitalize on heterosis by crossbreeding. After generations of crossbreeding and selection, Beefbooster cattle are now, for all intents and purposes, their own breed (although not registered). Beefbooster Inc. is located in Calgary, Alberta. Breed and population information is provided for each sequenced animal in Additional file 1.

For the seven purebred populations, animals were chosen for sequencing following an in-depth pedigree analysis performed using pedigree files obtained from the respective breed associations. Analysis of the pedigrees revealed good pedigree completeness and depth [1]. The programs CFC [2] and Pedig [3] were used to identify the most influential animals, and these were then ranked based on their total and marginal genetic contributions. Animals’ relationships with each other were also taken into account to avoid sequencing closely related animals. The top 30 ancestors were chosen for sequencing, with the goal of representing approximately 50 % of the effective genome. Younger bulls with a high number of progeny were also considered for sequencing. For the Alberta, Guelph, and Beefbooster populations, animals were selected based on number of progeny, and on relationships to animals with feed efficiency and meat quality measurements. To avoid duplication of sequencing efforts, animal selections were registered with the ongoing 1000 Bull Genomes Project [4].

The complete set of 379 genome sequences is described in Additional file 1, which includes the name, breed, and relevant population of each individual. An International Bull Identification (Interbull ID) number is also given to each animal. This ID consists of a three-letter breed code, followed by a three-letter country code, followed by a single letter to indicate the sex of the animal (M or F), and lastly a 12-character animal identifier. Thus the ID itself directly conveys country of registration, breed, and sex. The animal identifier can be used to retrieve additional animal information from various breed-specific databases. Identifiers for sequence retrieval from the NCBI Sequence Read Archive (SRA), and quality control measures and comments are also included in Additional file 1.

### Sequencing and sequence data quality assessment

The project used the SOLiD 5500xl system to sequence 85 animals, and the HiSeq 2000 platform for the remaining 294. Standard filtering criteria were used to remove low quality reads prior to alignment. Further quality checking was performed using FastQC version 0.10.1 [5]. Reads were mapped against the bovine genome assembly UMD 3.1 [6], including unassembled contigs using BWA version 0.5.9 [7]. Following read alignment, local realignment was performed using GATK version 2.4 [8], and duplicates were then marked using Picard version 1.54 [9]. Read mapping rate, duplication rate, and genome coverage without duplicate reads were determined and recorded for each sample (Additional file 1). The majority of samples yielded a high mapping rate (more than 95 % of reads mapped) and low duplicate reads rate (less than 10 %). IGV version 2.3 was used to visually inspect the read alignments for selected samples [10]. This visualization allowed us to identify and correct a software bug in the mapping pipeline for SOLiD paired-end data.

**Fig. 1:**
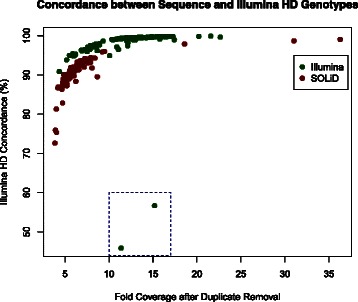
Concordance between sequence-derived genotypes and Illumina HD panel genotypes. Animals were sequenced using Illumina (*green*) or SOLiD (*red*) sequencing platforms. Sequence-derived genotypes were compared to those from the Illumina BovineHD BeadChip array (770 K) to determine concordance. Two animals (dashed box) showed unusually poor concordance. These animals (CHACANM000000FMC409 and HOLCANM000005429693) are flagged in Additional file 1

**Fig. 2:**
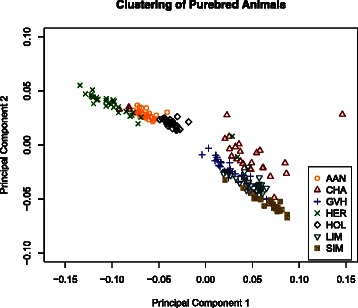
Principal component analysis of purebred animals based on sequence-derived genotypes. The genotypes of individuals from the seven purebred populations examined in this study were compared. Three Charolais samples appear in the Hereford cluster and two Hereford animals appear in the Charolais cluster, suggesting potential issues with sample sourcing or sample handling. These five samples are included in the final set of sequenced animals but are flagged as potentially problematic in Additional file 1. Angus (ANG), Charolais (CHA), Gelbvieh (GVH), Hereford (HER), Holstein (HOL), Limousin (LIM) and Simmental (SIM)

As a further data quality check, SNP calling was performed using Samtools-0.1.18 mpileup [11]. Sequencing genotypes were then compared to those obtained using the Illumina BovineHD BeadChip array (770 K) (Fig. 1) to establish the concordance rate. The median and mean SNP concordance rates for sequencing and genotyping are, respectively: 91.6 and 90.8 % for the SOLiD platform; and 99.4 and 98.2 % for the Illumina platform. This comparison allowed us to further assess data quality and to identify sample-handling errors. Finally, principal component analysis of genotypes from purebred animals was used to visualize the clustering of animals and to identify possible outliers (Fig. 2). Principal components 1 and 2 explain 75.9 % of the total variance. Quality control metrics and comments are included for each sequenced animal in Additional file 1.

## Availability of supporting data and materials

Sequence data is available from the NCBI SRA repository under BioProjects PRJNA176557 and PRJNA256210. Supporting data are also available from the GigaScience GigaDB database [12].

## Additional file

Additional file 1: **Bovine genome sequences from the Canadian Cattle Genome Project.** Description: Additional file 1 provides detailed information about each sequenced animal, including NCBI identifiers, Interbull ID, animal name, breed, population, sequencing platform, sequencing depth, and concordance rate. (XLSX 99 kb)

## Abbreviations

BWA: Burrows-wheeler aligner
CFC: Coancestry, inbreeding (F) and contribution
GATK: Genome analysis toolkit
IGV: Integrative genomics viewer
KC: Kinsella composite
SNP: Single nucleotide polymorphism
UMD: University of Maryland
